# Efficacy and Safety of Immune Checkpoint Blockades in the Treatment of Ocular Melanoma: A Systematic Review and Meta-Analysis

**DOI:** 10.3389/fonc.2021.781162

**Published:** 2021-12-06

**Authors:** Lu Zhao, Wenwen Xia, Yan Zhang, Peng Zou, Qiwen Zhu, Rong Zhang

**Affiliations:** ^1^ Department of Biopharmaceutics, School of Life Science and Bio-Pharmaceutics, Shenyang Pharmaceutical University, Shenyang, China; ^2^ Department of Biochemistry and Molecular Biology, School of Basic Medical Sciences, Shenyang Medical College, Shenyang, China; ^3^ School of Traditional Chinese Medical, Shenyang Medical College, Shenyang, China; ^4^ Key Laboratory of Behavioral and Cognitive Neuroscience of Liaoning Province, Shenyang Medical College, Shenyang, China

**Keywords:** ocular melanoma, immune checkpoint blockade, PD-1, PD-L1, CTLA-4, meta-analysis

## Abstract

**Aim:**

This meta-analysis aimed to compare the efficacy and safety of immune checkpoint blockade for the treatment of ocular melanoma.

**Methods:**

We searched the PubMed, EMBASE, and Cochrane Library databases up to July 2021. Effect sizes (ESs) and corresponding 95% confidence intervals (CIs) were used to compare the outcomes. Efficacy outcomes included complete response (CR), partial response (PR), stable disease (SD), progressive disease (PD), objective response rate (ORR), overall survival (OS), progression free survival (PFS). Safety outcomes included adverse events (AEs) and serious adverse event (SAEs).

**Results:**

A total of 16 eligible articles with 848 ocular melanoma patients were included. ICB treatment significantly improved CR (ES=0.02, 95%CI: 0.00-0.03, P=0.023), PR (ES=0.07, 95%CI: 0.05-0.09, P=0.000), SD (ES=0.31, 95%CI: 0.17-0.46, P=0.000), PD (ES=0.69, 95%CI: 0.61-0.77, P=0.000), ORR (ES=0.10, 95%CI: 0.04-0.15, P=0.000), OS (ES=9.68, 95%CI: 7.28-12.07, P=0.000) and PFS (ES=2.88, 95%CI: 2.69-3.07, P=0.000) in patients with ocular melanoma. Moreover, ICB therapies were associated with reduced AEs (ES=0.48, 95%CI: 0.30-0.67, P=0.000) and SAEs (ES=0.31, 95%CI: 0.18-0.45, P=0.000).

**Conclusions:**

ICB therapy showed good efficacy and safety in treating patients with ocular melanoma.

## Introduction

Uveal melanoma (UM) is the most common primary ocular malignancy in adults. It originates from melanocytes of the iris, ciliary body, and choroid (1–3). In Europe, the incidence of UM is 4–7 cases/million, and is much rarer than cutaneous melanoma. In a study in 2011, the incidence of uveal melanoma in the US was reported to be 5.1 per million (4). Although UM accounts for <5% of all cases in the United States; however, it remains to be the most common primary ocular malignancy in adults in the US and accounts for 85–95% of all cases of ocular melanoma (5).

Many approaches exist to treat primary UM with the aim of reducing tumor growth and even preserving vision in the affected eye. These approaches include local management by globe-preserving therapy such as brachytherapy, radiotherapy, laser, or surgical resection, or enucleation (5). In 2006, a Collaborative Ocular Melanoma Study Group (COMS) demonstrated that UM patients with medium-sized choroidal melanomas treated with either iodine-125 brachytherapy or enucleation demonstrated equivalent survival outcomes (6). In the US, the majority of patients with primary uveal melanomas are treated with first-line plaque brachytherapy.

Despite effective local therapies, nearly 40–50% of UM patients will ultimately develop distant metastasis (7, 8). Up to 95% of cases of metastatic UM spread to the liver (2, 9, 10). The median survival of UM patients who have developed liver metastasis is 6–12 months (11–13).

In recent years, treatment with immune checkpoint blockade (ICB) with programmed death 1 (PD-1) inhibitors as well as ipilimumab, which is an anti-cytotoxic T lymphocyte-associated antigen (CTLA-4) antibody, have yielded promising results for the treatment of cutaneous melanoma (CM) (14–17). However, thus far, no pivotal trials have investigated the efficacy of these treatments in UM.

Currently, immune checkpoint blockade (ICB), which has been successfully used in cutaneous melanoma, is increasingly being adopted for treating UM. In recent years, blocking immune checkpoint proteins such as PD-1/PD-L1 and CTLA-4 have emerged as a pivotal treatment for melanoma patients, which is associated with strong survival benefits (14, 15, 18). Previous meta-analyses have shown that UM is less responsive to ipilimumab therapy regardless of dosage (19). Therefore, the usefulness of ICB in ocular melanoma requires additional investigation. In the present meta-analysis, we evaluated the efficacy and safety of ICB for the treatment of ocular melanoma.

## Materials and Methods

We performed a meta-analysis in accordance with the Preferred Reporting Items for Systematic Reviews and Meta-Analyses (PRISMA2020) guidelines. We started by searching relevant articles by the patients, intervention, comparator, outcomes, and study design (PICOS) principle, and the articles were then screened for inclusion and exclusion criteria.

### Eligibility Criteria

The inclusion criteria included the following: 1) patients who were diagnosed as having ocular melanoma; 2) who received ICB interventions (anti-PD-1/PD-L1 antibodies, anti-CTLA-4 antibodies, PD-1 inhibitors); 3) had efficacious outcomes including complete response (CR), partial response (PR), stable disease (SD), progressive disease (PD), objective response rate (ORR), overall survival (OS), progression-free survival (PFS), and disease control rate (DCR); safety outcomes including adverse events (AEs), serious adverse event (SAEs); 4) study types including cohort and single-arm studies.

The exclusion criteria were as follows: 1) studies that were in the form of conference abstracts, case reports, meta-analysis or review, animal study, and protocol; 2) those that had been written in languages other than English; and 3) studies whose full text could not be obtained or unavailable data.

### Search Strategy

We started a systematic search on the PubMed, EMBASE, and Cochrane Library databases from inception to July 2021 for potentially eligible studies, using the MeSH terms ‘ocular melanoma’ and ‘immune checkpoint inhibitors’ and relevant key words.

### Data Extraction and Quality Assessment

The selection and inclusion of studies were performed in two stages by two independent reviewers (LZ and WX). This included the analysis of titles/abstracts followed by the full texts. Disagreements were resolved by a third reviewer (RZ).

The data retrieved included the names of authors, publication year, study design, country; patient characteristics (number of patients, age, gender, sample size, intervention, dosage, follow-up time, and outcomes).

The methodological quality of the cohort and single-arm studies was evaluated using the Newcastle and Ottawa Scale (NOS) (20), with the maximum possible score of 9 points representing the least risk of bias. Quality assessment was performed in duplicate by 2 researchers separately (LZ and WX).

### Statistical Analysis

Effect sizes (ESs) and corresponding 95% confidence intervals (CIs) were used to compare the outcomes. Cochran’s Q statistic (P < 0.10 indicated evidence of heterogeneity) assessed the heterogeneity among studies (21). When significant heterogeneity (P < 0.10) was detected, the random-effects model was used to combine the effect sizes of the included studies. Otherwise, the fixed-effects model was adopted. (Higgins, J.P.T. and Green, S. (2011) Cochrane handbook for systematic reviews of interventions version 5.1.0. Naunyn Schmied. Arch. Exp. Pathol. Pharmakol. 5, S38) (22). All analyses were performed using STATA SE software version 14.0 (StataCorp, College Station, Texas, USA).

## Results

### Identification of Eligible Studies

**Figure 1:**
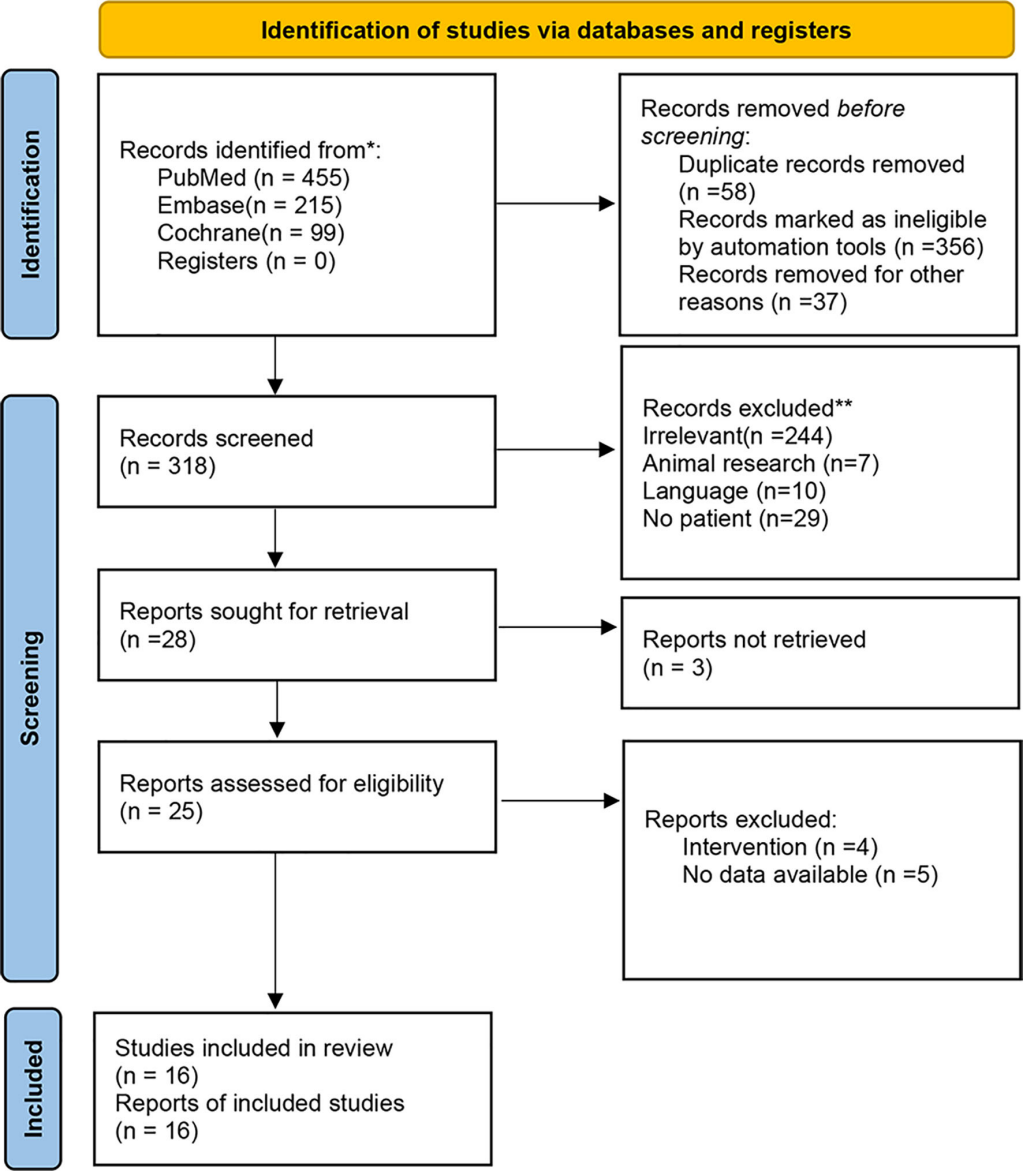
Consort chart of the included/excluded studies.

A total of 769 relevant publications were initially identified *via* databases and registers. Duplicates and other ineligible records defined by automation tools were removed. The remaining 318 publications were screened for eligibility. Due to insufficient data information, non-human research, and language restriction, 302 documents were further excluded. Finally, 16 full-text articles were included for this meta-analysis. **Figure 1** is a flowchart illustrating the screening process.

### Study Characteristics and Quality Assessment

**Table 1** describes the characteristics of the 16 eligible articles (23–38) consisting of 13 cohort and 3 single-arm studies. A total of 848 ocular melanoma patients were enrolled, with an age range of 16–65 years. The proportion of male patients ranged from 29.4% to 63.6%. Interventions included anti-PD-1-antibodies (nivolumab, pembrolizumab), anti-CTLA-4 antibodies (ipilimumab, tremelimumab), anti-PD-L1 antibodies, and PD-1 inhibitor. The follow-up time ranged from 12–96 weeks. The treatment outcomes included complete response (CR), partial response (PR), stable disease (SD), progressive disease (PD) and objective response rate (ORR), overall survival (OS), and progression-free survival (PFS).

**Table 1:** List of included studies.

Author	Design	Sample size	Age	Male %	Intervention	Dosage (mg/kg)	Follow-up (weeks)	Outcome
Algazi (23)	Cohort	56	62.4	32 (57.1%)	PD-1+PD-L1 antibodies	/	12w	ORR/PFS/OS/AE
Bol (24)	Cohort	126	pre62/post65	Pre 14 (43.8%)/post 47 (50%)	Pembrolizumab + nivolumab + ipilimumab	/	92w	CR/PR/SD/PD
Del Vecchio et al. (25)	Cohort	71	63	27(38%)	Ipilimumab	3 mg/kg	87.2w	PFS/OS/AE
Heppt (26)	Cohort	64	< 60 years-28/≥ 60 years-36	33 (51.6%)	Ipilimumab+PD-1 inhibitor	3 mg/kg or 1 mg/kg	12w	PFS/OS/AE/CR/PR/SD/PD/ORR
Heppt (27)	Cohort	96	/	/	Ipilimumab+PD-1 inhibitor	3 mg/kg or 2 mg/kg	12w	AE/CR/PR/SD/PD
Joshua (28)	Cohort	11	58	7 (63.6%)	Tremelimumab	15 mg/kg	44w	PFS/OS/AE
Kelderman (29)	Cohort	22	>16	/	Ipilimumab	3 mg/kg	12w	PFS/OS/AE/CR/PR/SD/PD
Maio (30)	Cohort	83	62	39 (47%)	Ipilimumab	3 mg/kg	22.4w	PFS/OS/AE
Najjar (31)	Cohort	89	53	47 (53%)	Ipilimumab + nivolumab	/	36.8w	PFS/OS/AE/CR/PR/SD/PD/ORR
Pelster (32)	Single-ar m	35	62	12 (34%)	Ipilimumab + nivolumab	3 mg/kg + 1 mg/kg	96w	PFS/OS/AE/CR/PR/SD/PD/ORR
Piulats (33)	Single-arm	52	59.1	29 (55.8%)	Ipilimumab + nivolumab	3 mg/kg + 1 mg/kg	48w	PFS/OS/AE/CR/PR/SD/PD
Rossi (34)	Cohort	17	63.8	9 (52.9%)	Pembrolizumab	2 mg/kg	/	PFS/AE/CR/PR/SD/PD
Rozeman (35)	Cohort	19	63	12 (63%)	Ipilimumab	3 mg/kg	12w	AE/CR/PR/SD/PD
Sander et al. (36)	Cohort	37	59.2	21 (56.8%)	Pembrolizumab + nivolumab + ipilimumab	2 mg/kg+3 mg/kg+(3 mg/kg + 1 mg/kg)	47.2w	OS/AE/CR/PR/SD/PD
Van der Kooij (37)	Cohort	17	56.9	5 (29.4%)	Pembrolizumab + nivolumab	2 mg/kg or 3 mg/kg	16w	PFS/OS/AE
Zimmer (38)	Single-arm	53	67	23 (43%)	Ipilimumab	3 mg/kg	48w	PFS/OS/AE/CR/PR/SD/PD/DCR

ORR, objective response rate; CR, complete response; PR, partial response; SD, Stable disease; PD, progressive disease; PFS, progression Free Survival; OS, overall Survival; AE, adverse event; DCR, disease control rate.

NOS analysis deemed all 13 cohort studies to be of high methodological quality. Two single-arm studies scored 12, and one scored 13 according to the Methodological Index for Non-Randomized Studies (MINORS), indicating medium quality (**Table 2**).

**Table 2:** Quality assessment of included studies.

Study (NOS)	Representativeness of the Exposed Cohort	Selection of the Non-Exposed Cohort	Ascertainment of Exposure	Demonstration That Outcome of Interest Was Not Present at Start of Study	Comparability of Cohorts on the Basis of the Design or Analysis	Assessment of Outcome	Was Follow-Up Long Enough for Outcomes to Occur	Adequacy of Follow Up of Cohorts
Algazi (23)	*	*	*	*	*	*	*	*
Bol (24)	*	*	*	*	**	*	*	*
Del Vecchio et al. (25)	*	*	*	*	**	*	*	*
Heppt (26)	*	*	*	*	**	*	*	*
Heppt (27)	*	*	*	*	**	*	*	*
Joshua (28)	*	*	*	*	*	*	*	*
Kelderman (29)	*	*	*	*	*	*	*	*
Maio (30)	*	*	*	*	*	*	*	*
Najjar (31)	*	*	*	*	*	*	*	*
Rossi (34)	*	*	*	*	*	*	*	*
Rozeman (35)	*	*	*	*	*	*	*	*
Sander et al. (36)	*	*	*	*	**	*	*	*
Van der Kooij (37)	*	*	*	*	*	*	*	*

*Item score.

### CR, PR, SD, PD, and ORR Outcomes

**Figure 2:**
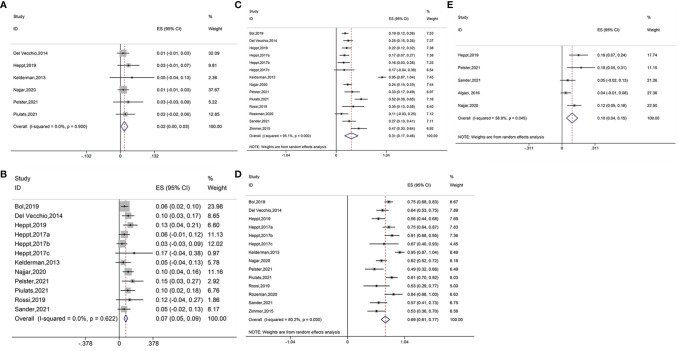
**(A)** Forest plot of complete response (CR) with immune checkpoint blockade treatment. **(B)** Forest plot of partial response (PR) with immune checkpoint blockade treatment. **(C)** Forest plot of stable disease (SD) with immune checkpoint blockade treatment. **(D)** Forest plot of progressive disease (PD) with immune checkpoint blockade treatment. **(E)** Forest plot of objective response rate (ORR) with immune checkpoint blockade treatment.

Six studies reported the outcome of CR in ICB treatment for ocular melanoma. The pooled results suggested that immune checkpoint blockage significantly improved CR in patients with ocular melanoma (ES = 0.02, 95% CI: 0.00–0.03, P = 0.023; I^2^ = 0.0%, P_heterogeneity_ = 0.900) (**Figure 2A**). Subgroup analysis showed no significant increase in the CR between ipilimumab (ES = 0.01, 95% CI: –0.01–0.04, P = 0.284; I^2^ = 0.0%, P_heterogeneity_ = 0.445), ipilimumab plus PD-1 inhibitor (ES = 0.03, 95% CI: –0.01–0.07, P = 0.152);, and ipilimumab plus nivolumab (ES = 0.01, 95% CI: –0.00–0.03, P = 0.108; I^2^ = 0.0%, P_heterogeneity_ = 0.807). No heterogeneity was detected between groups (P_heterogeneity_ = 0.743, **Figure S1A**).

Twelve studies reported PR results. Pooled analysis suggested that the immune interventions significantly improved the PR (ES = 0.07, 95% CI: 0.05–0.09, P=0.000; I^2^ = 0.0%, P_heterogeneity_ = 0.622) (**Figure 2B**). Subgroup analysis showed significant benefits in the PR for treatments using pembrolizumab plus nivolumab and pilimumab (ES = 0.06, 95%CI: 0.02–0.09, P = 0.002; I^2^ = 0.0%, P_heterogeneity_ = 0.907), ipilimumab (ES = 0.08, 95%CI: 0.02–0.13, P = 0.005; I^2^ = 0.0%, P_heterogeneity_ = 0.335), ipilimumab plus PD-1 inhibitor (ES = 0.13, 95% CI: 0.05–0.21, P = 0.001; I^2^ = 0.0%,P_heterogeneity_ = 0.716), pembrolizumab (ES = 0.07, 95% CI: 0.01–0.12, P = 0.026; I^2^ = 0.0%, P_heterogeneity_ = 0.476), and ipilimumab plus nivolumab (ES = 0.11, 95% CI: 0.06–0.15, P = 0.000; I^2^ = 0.0%, P_heterogeneity_ = 0.742). The use of nivolumab alone did not increase the PR (ES = 0.03, 95% CI: –0.03–0.09, P = 0.312). The heterogeneity across different groups was not significant (P_heterogeneity_ = 0.235, **Figure S1B**).

Fourteen studies reported the data of SD. Overall, significant improvement in SD was observed for ocular melanoma (ES = 0.31, 95% CI: 0.17–0.46, P = 0.000; I^2^ = 95.1%, P_heterogeneity_ = 0.000) (**Figure 2C**). The SD was also found to increase when using various immune checkpoint inhibitors including pembrolizumab with nivolumab and ipilimumab (ES = 0.20, 95% CI: 0.14–0.27, P = 0.000; I^2^ = 6.3%, P_heterogeneity_ = 0.302), ipilimumab (ES = 0.45, 95% CI: 0.02–0.87, P = 0.038; I^2^ = 98.1%, P_heterogeneity_ = 0.000), ipilimumab plus PD-1 inhibitor (ES = 0.21, 95% CI: 0.12–0.30, P = 0.000; I^2^ = 0.0%, P_heterogeneity_ = 0.663), pembrolizumab (ES = 0.23, 95% CI: 0.06–0.40, P = 0.007; I^2^ = 51.9%,P_heterogeneity_ = 0.149), nivolumab (ES = 0.16, 95% CI: 0.03–0.28, P = 0.015), and ipilimumab plus nivolumab (ES = 0.36, 95% CI: 0.19–0.53, P = 0.000; I^2^ = 82.4%, P_heterogeneity_ = 0.003) (**Figure S1C**).

Fourteen studies reported PD outcome. Results of the pooled analysis revealed that treatment with ICB improved the PD (ES = 0.69, 95% CI: 0.61–0.77, P = 0.000; I^2^ = 80.2%, P_heterogeneity_ = 0.000) (**Figure 2D**). Similar benefits were shown for the PD when using treatments of pembrolizumab combined with nivolumab and ipilimumab (ES = 0.67, 95% CI: 0.49–0.85, P = 0.000; I^2^ = 76.3%, P_heterogeneity_ = 0.040), ipilimumab (ES = 0.67, 95% CI: 0.51–0.83, P = 0.000; I^2^ = 71.4%, P_heterogeneity_ = 0.030), ipilimumab plus PD-1 inhibitor (ES = 0.58, 95% CI: 0.47–0.69, P=0.000; I^2^ = 0.0%, P_heterogeneity_ = 0.487), pembrolizumab (ES = 0.67, 95% CI: 0.45–0.88, P = 0.000; I^2^ = 64.2%, P_heterogeneity_ = 0.095), nivolumab (ES = 0.89, 95%CI: 0.76–1.03, P = 0.000; I^2^ = 66.8%, P_heterogeneity_ = 0.083), and ipilimumab combined with nivolumab (ES = 0.65, 95% CI: 0.48–0.82, P = 0.000; I^2^ = 82.7%, P_heterogeneity_ = 0.003) (**Figure S1D**).

Pooled analysis of five studies suggested beneficial ORR outcome (ES = 0.10, 95% CI: 0.04–0.15, P = 0.000; I^2^ = 58.9%, P_heterogeneity_ = 0.045) (**Figure 2E**). The ORR was also found to increase when using ipilimumab plus PD-1 inhibitor (ES = 0.16, 95% CI: 0.07–0.24, P = 0.000) and ipilimumab combined with nivolumab (ES = 0.13, 95% CI: 0.07–0.19, P = 0.000; I^2^ = 0.0%, P_heterogeneity_ = 0.395). However, no improvements were seen in the ORR when using pembrolizumab in combination with nivolumab and ipilimumab (ES = 0.05, 95%CI: –0.02–0.13, P = 0.146), PD-1, and PD-L1 antibodies (ES = 0.04, 95% CI: –0.01–0.08, P = 0.148) (**Figure S1E**).

### OS and PFS Outcomes

**Figure 3:**
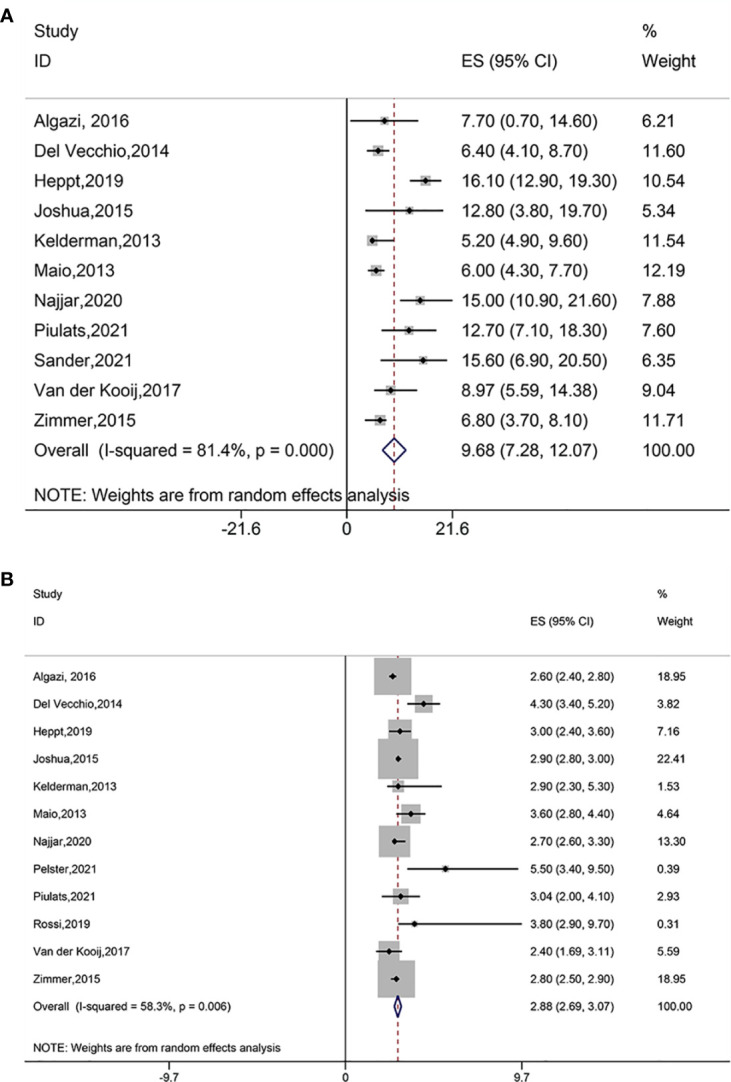
**(A)** Forest plot of overall survival (OS) of immune checkpoint blockade treatment. **(B)** Forest plot of progression free survival (PFS) of immune checkpoint blockade treatment.

Pooled analysis including eleven studies suggested favorable OS outcome (ES = 9.68, 95% CI: 7.28–12.07, P = 0.000; I^2^ = 81.4%, P_heterogeneity_ = 0.000) (**Figure 3A**). Subgroup analysis showed similar OS benefits when treatments used PD-1 and PD-L1 antibodies (ES=7.70, 95%CI: 0.75–14.65, P = 0.030), ipilimumab (ES = 6.10, 95%CI: 5.06–7.15, P = 0.000; I^2^ = 0.0%, P_heterogeneity_ = 0.794), ipilimumab plus PD-1 inhibitor (ES = 16.10, 95% CI: 12.90–19.30, P = 0.000), tremelimumab (ES = 12.80, 95% CI: 4.85–20.75, P = 0.002), ipilimumab plus nivolumab (ES=13.90, 95%CI: 10.03–17.77, P=0.000; I^2^ = 0.0%, P_heterogeneity_ = 0.561), pembrolizumab plus nivolumab plus ipilimumab (ES = 15.60, 95% CI: 8.80–22.40, P = 0.000), and pembrolizumab plus nivolumab (ES = 8.97, 95% CI: 4.58–13.37, P = 0.000) (**Figure S2A**).

Pooled results with twelve studies suggested favorable PFS outcome (ES = 2.88, 95% CI: 2.69–3.07, P = 0.000; I^2^ = 58.3%, P_heterogeneity_ = 0.006) (**Figure 3B**). The PFS was found to improve in treatments using PD-1 and PD-L1 antibodies (ES = 2.60, 95% CI: 2.40–2.80, P = 0.000), ipilimumab (ES = 3.38, 95%CI: 2.60–4.16, P = 0.000; I^2^ = 77.2%, P_heterogeneity_ = 0.004), ipilimumab plus PD-1 inhibitor (ES = 3.00, 95% CI: 2.40–2.60, P = 0.000), tremelimumab (ES = 2.90, 95% CI: 2.80–3.00, P = 0.000), ipilimumab plus nivolumab (ES = 2.97, 95% CI: 2.20–3.74, P = 0.000; I^2^ = 42.6%, P_heterogeneity_ = 0.175), pembrolizumab in combination with nivolumab and ipilimumab (ES = 3.80, 95% CI: 0.40–7.20, P = 0.028), and pembrolizumab plus nivolumab (ES = 2.40, 95% CI: 1.69–3.11, P = 0.000) (**Figure S2B**).

### Safety Outcomes

**Figure 4:**
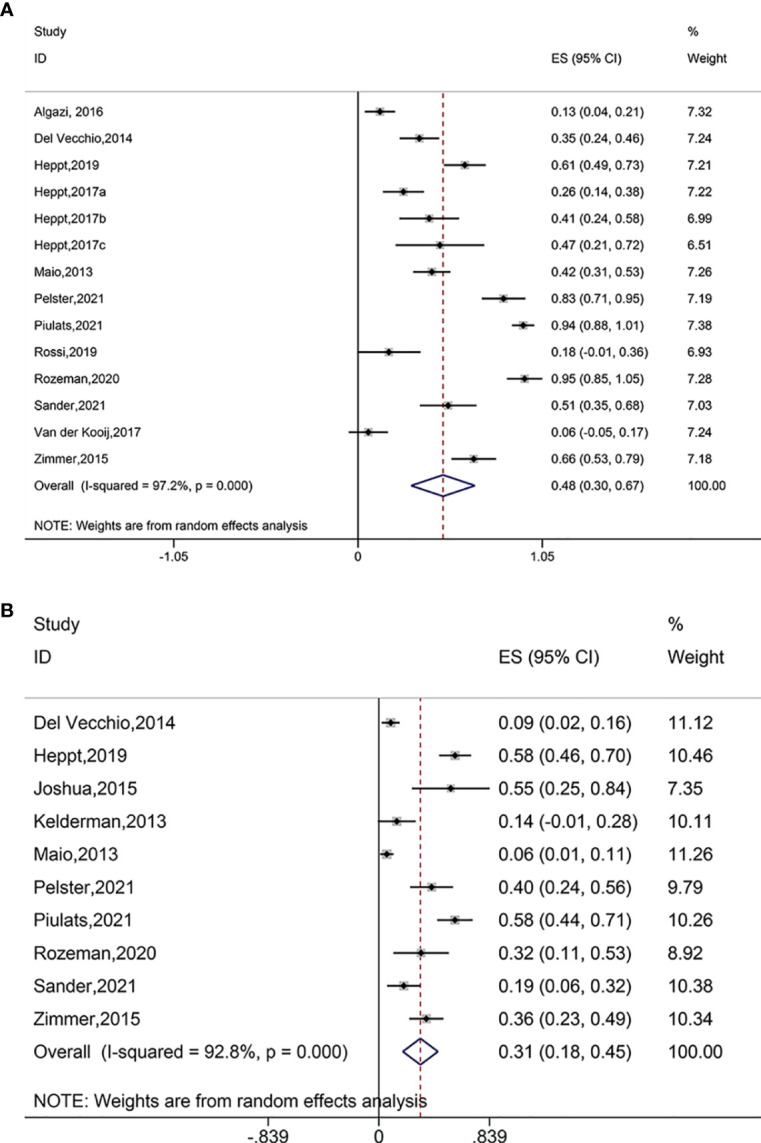
**(A)** Forest plot of adverse events (AEs) after treatment with immune checkpoint blockade. **(B)** Forest plot of serious adverse event (SAEs) after treatment with immune checkpoint blockade.

Pooled analysis of fourteen studies suggested significantly reduced AEs after treatment with immune checkpoint blockade (ES = 0.48, 95% CI: 0.30–0.67, P = 0.000; I^2^ = 97.2%, P_heterogeneity_ = 0.000) (**Figure 4A**). The AEs also decreased when using PD-1 and PD-L1 antibodies (ES = 0.13, 95% CI: 0.04–0.21, P= 0.000), ipilimumab (ES = 0.60, 95% CI: 0.31–0.88, P = 0.000; I^2^ = 96.1%, P_heterogeneity_ = 0.000), ipilimumab plus PD-1 inhibitor (ES = 0.58, 95% CI: 0.47–0.69, P = 0.000; I^2^ = 0.0%, P_heterogeneity_ = 0.319), pembrolizumab (ES = 0.23, 95% CI: 0.14–0.33, P = 0.000; I^2^ = 0.0%, P_heterogeneity_ = 0.450), nivolumab (ES = 0.41, 95% CI: 0.24–0.58, P = 0.000);, ipilimumab plus nivolumab (ES = 0.90, 95% CI: 0.79–1.01, P = 0.000; I^2^ = 59.5%, P_heterogeneity_=0.116), and pembrolizumab in combination with nivolumab and ipilimumab (ES = 0.51, 95% CI: 0.35–0.68, P = 0.000). The AEs did not significantly reduce when using pembrolizumab plus nivolumab (ES = 0.06, 95% CI: –0.05–0.17, P = 0.302) (**Figure S3A**). Furthermore, all treatment modalities investigated were associated with significantly reduced AEs and SAEs.

Pooled analysis of ten studies suggested significantly reduced SAEs after immune checkpoint blockade treatments (ES = 0.31, 95% CI: 0.18–0.45, P = 0.000; I^2^ = 92.8%, P_heterogeneity_ = 0.000) (**Figure 4B**). Subgroup analysis showed decreased SAEs when using ipilimumab (ES = 0.17, 95% CI: 0.07–0.28, P = 0.001; I^2^ = 82.1%, P_heterogeneity_ = 0.000), ipilimumab plus PD-1 inhibitor (ES = 0.58, 95% CI: 0.46–0.70, P = 0.000), tremelimumab (ES = 0.55, 95% CI: 0.25–0.84, P = 0.000), ipilimumab plus nivolumab (ES = 0.49, 95% CI: 0.32–0.67, P = 0.000; I^2^ = 63.1%, P_heterogeneity_ = 0.100), and pembrolizumab in combination with nivolumab and ipilimumab (ES = 0.19, 95% CI: 0.06–0.32, P = 0.003) (**Figure S3B**).

### Sensitivity Analysis

Sensitivity analysis showed that certain individual study exclusions could influence the overall estimated effects of AE, PD, SAE, and SD, without affecting the OS, PFS, and PR (**Figure S6**).

### Publication Bias

The Begg test for funnel plot asymmetry indicated the presence of significant publication bias for PR (P = 0.043) and SAE (P = 0.015). No significant publication bias was detected for the AE (P = 0.190), OS (P = 0.551), PD (P = 0.077), PFS (P = 0.250), and SD (P = 0.824; **Figure S5**).

## Discussion

This meta-analysis systematically evaluated the efficacy and safety of immune checkpoint blockade in the treatment of patients with ocular melanoma. The results showed that immune checkpoint blockade significantly improved the outcomes of CR, PR, SD, PD, ORR, OS, and PFS in ocular melanoma patients. In addition, immune checkpoint blockade was associated with reduced AEs and SAEs.

This meta-analysis included cohort and phase-II single-arm studies investigating the effect of ICB treatment for ocular melanoma. Immune-related interventions covered different immune checkpoint inhibitors. Six studies investigated CTLA-4 blockade monotherapy with ipilimumab (at 3 mg/kg) or tremelimumab (at 15 mg/kg), and one study investigated PD-1 blockade monotherapy (pembrolizumab at 2 mg/kg). Additionally, seven studies investigated the combination of CTLA4 and PD-1 blockade, including PD-1 inhibitors or anti-PD-1 antibodies. Combined anti-PD-1 antibodies including pembrolizumab and nivolumab, and anti-PD-1 plus anti-PD-L1 antibodies were also evaluated in one study, respectively. This meta-analysis showed that all treatment modalities significantly improved the SD, PD, OS, and PFS of patients regardless of immune checkpoint inhibitor types, indicating stable disease and long-term survival benefits. Subgroup analysis revealed that CTLA-4 blockade monotherapy (ipilimumab), combined CTLA-4 and PD-1 blockade (ipilimumab with Nivolumab or PD-1 inhibitor) did not improve the CR of patients. The monotherapy with anti-PD-1 antibody nivolumab did not increase PR. Additionally, combined PD-1 and CTLA-4 blockade using pembrolizumab, nivolumab and ipilimumab did not lead to an ORR benefit. These results suggested that, compared with monotherapy, the combination of CTLA-4 and PD-1 blockade might be more effective on the clinical response of PR, but not on the CR and ORR for ocular melanoma patients. Furthermore, immune checkpoint inhibitor therapies were associated with significantly reduced AEs and SAEs, suggesting a good safety profile for 4ICB treatment.

A previous review by Heppt et al. suggested that ICB had lower efficacy in the treatment of advanced uveal melanoma (UM) patients and maintained a high toxicity profile (19). The results showed that UM patients exhibited a minimal response to CTLA-4 blockade monotherapy (ipilimumab or tremelimumab) in terms of the ORR, PFS, and OS. Anti-PD-1 antibodies including pembrolizumab (2 mg/kg) and nivolumab (3 mg/kg) yielded an ORR of 30% and 6%, respectively. No treatment response was observed with CTLA-4 and PD-1 combined blockade, and the median PFS was 2.9 months. Their study comprised of 12 records from 7 expanded access programs (EAP) or named patient programs (NPP), 4 phase II trials, and 1 phase Ib trial for a qualitative synthesis, and no RCT was found. Conference abstracts with incomplete or preliminary data were also included. Therefore, there was a high selection bias in that review. The effects of mixed response on outcomes were reported but could not be combined for a quantitative analysis.

Our meta-analysis also demonstrated the favorable outcome of disease control rate (DCR) following ICB treatment (**Figure S4**). Vecchio et al. reported immune-related disease control for 36% of patients with metastatic mucosal melanoma, and in most cases, the improved DCR was attributed to SD rather than to a significant reduction in the tumor burden (36). Sander et al. (36) showed that beneficial ORR was notable for metastatic UM patients who achieved a PR, and patients having attained either a PR or SD had an improved DCR (36). Moreover, though a modified immune prognostic index (IPI) status might be used to predict a survival benefit from ICB treatment, the IPI score was not found to be associated with ORR and DCR. The most likely explanation was that the statistical power was sufficient for OS analysis, rather than ORR and DCR assessments. Favorable ORR (defined as CR +PR) and DCR (defined as SD +CR+PR) with ICB in treating metastatic UM have also been reported (38, 39).

The present study has several noted limitations. First, due to the retrospective and uncontrolled design, the results should be interpreted with caution. Secondly, some of the included studies had a relatively small sample size, which would produce an overestimate of the treatment effect when compared with larger studies. Third, no RCTs were identified in this study. The current evidence supporting the application of ICB in UM still needs to be verified in the more rigorously designed RCTs.

## Conclusion

This meta-analysis showed that ICB was effective in treating ocular melanoma patients in terms of patients’ clinical responses and long-term survival, with a good safety profile. Further larger-scale more rigorously studies are warranted to confirm our findings.

## Data Availability Statement

The original contributions presented in the study are included in the article/**Supplementary Material**. Further inquiries can be directed to the corresponding authors.

## Author Contributions

LZ: Methodology, Formal Analysis, Resources, Writing Original Draft Preparation, Writing Review and Editing. WX: Software, Data Curation. YZ: Conceptualization. PZ: Investigation. QZ: Validation, Visualization, Supervision. RZ: Project Administration, Funding Acquisition. All authors contributed to the article and approved the submitted version.

## Funding

This work was supported by the National Natural Science Foundation of China [Grant numbers: 31772518]; Innovation Team and Talent Support Programs of Colleges in Liaoning Province [Grant numbers: LT2019013 and LR2019071]; Support Plan of Shenyang Pharmaceutical University for Youth Development [Grant Number: ZQN2014A05]; Natural Science Foundation of Liaoning Province [Grant Number: 2019-MS-295].

## Conflict of Interest

The authors declare that the research was conducted in the absence of any commercial or financial relationships that could be construed as a potential conflict of interest.

## Publisher’s Note

All claims expressed in this article are solely those of the authors and do not necessarily represent those of their affiliated organizations, or those of the publisher, the editors and the reviewers. Any product that may be evaluated in this article, or claim that may be made by its manufacturer, is not guaranteed or endorsed by the publisher.
