# Supplementary material for: Efficacy and Safety of Immune Checkpoint Blockades in the Treatment of Ocular Melanoma: A Systematic Review and Meta-Analysis
Source: Front Oncol. 2021 Dec 6;11:781162. doi: 10.3389/fonc.2021.781162 (PMC8685375; doi:10.3389/fonc.2021.781162)
Supplement: Supplementary file 1 [file DataSheet_1.docx]

Supplementary Material

## Supplementary Figures

**Figure S1.**


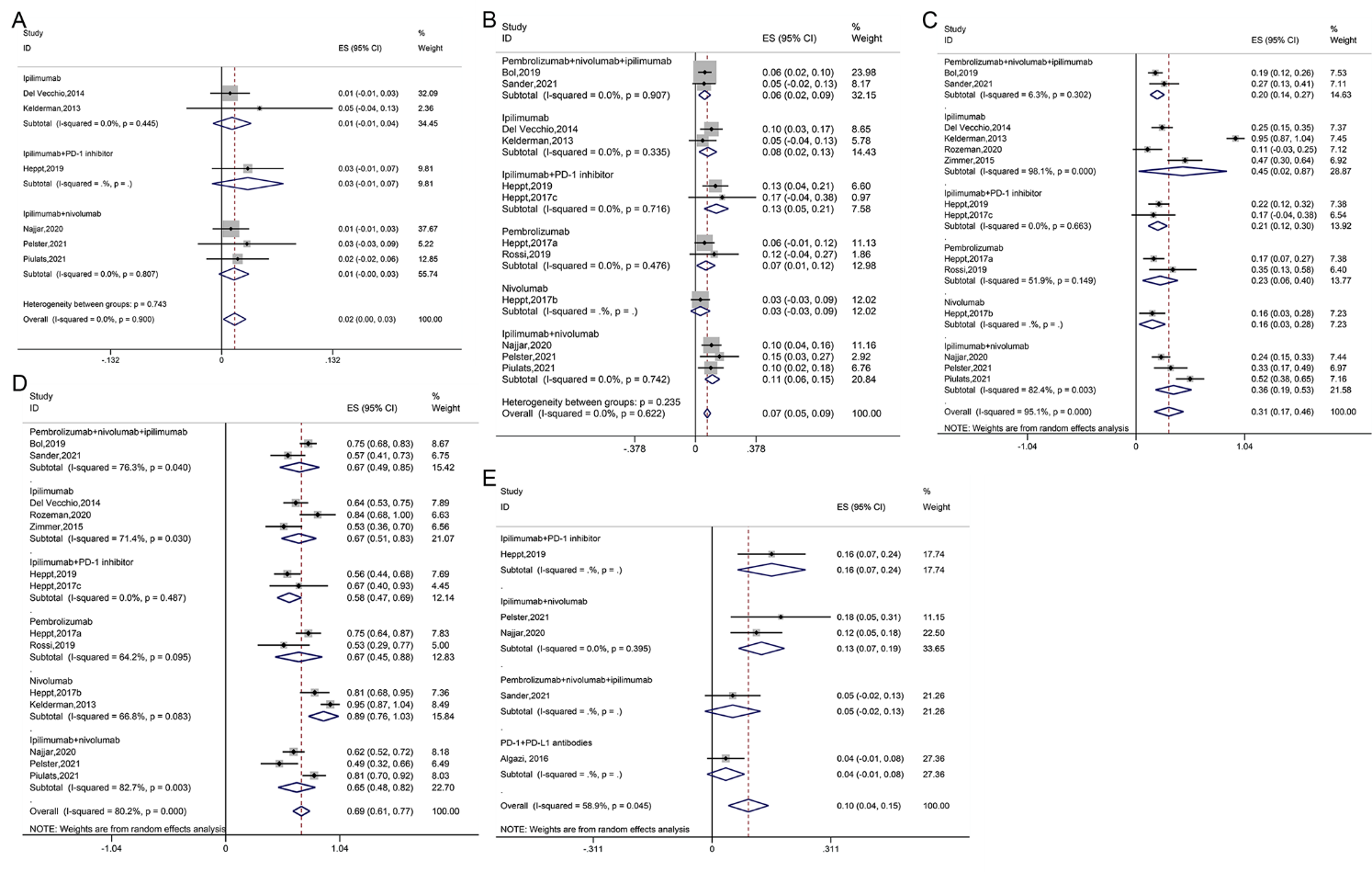


(A) Forest plot of complete response (CR) with immune checkpoint blockade treatment about subgroup analysis. (B) Forest plot of partial response (PR) with immune checkpoint blockade treatment about subgroup analysis. (C) Forest plot of stable disease (SD) with immune checkpoint blockade treatment about subgroup analysis. (D) Forest plot of progressive disease (PD) with immune checkpoint blockade treatment about subgroup analysis. (E) Forest plot of objective response rate (ORR) with immune checkpoint blockade treatment about subgroup analysis.

**Figure S2.**


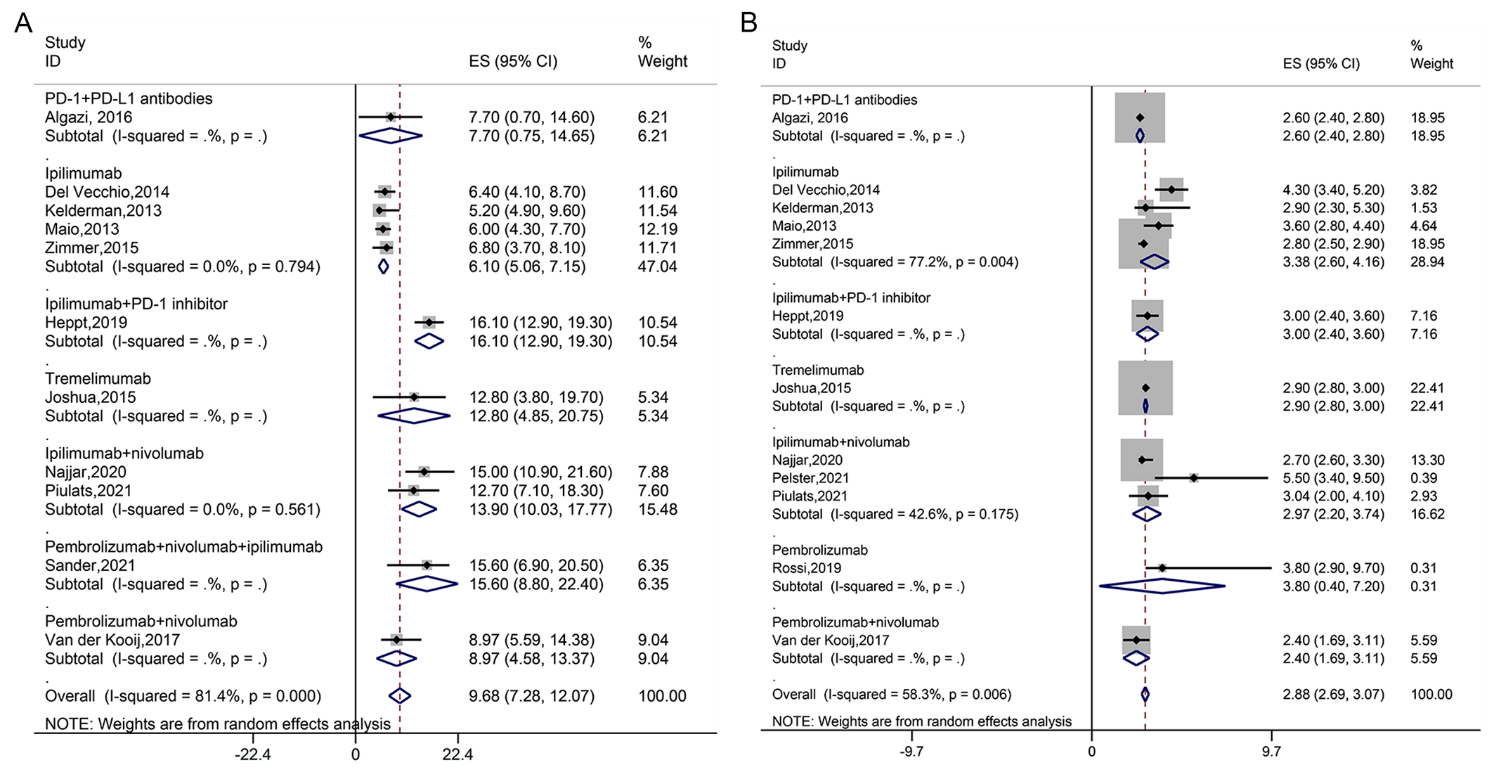


(A) Forest plot of overall survival (OS) of immune checkpoint blockade treatment about subgroups analysis. (B) Forest plot of progression free survival (PFS) of immune checkpoint blockade treatment about subgroups analysis.

**Figure S3.**


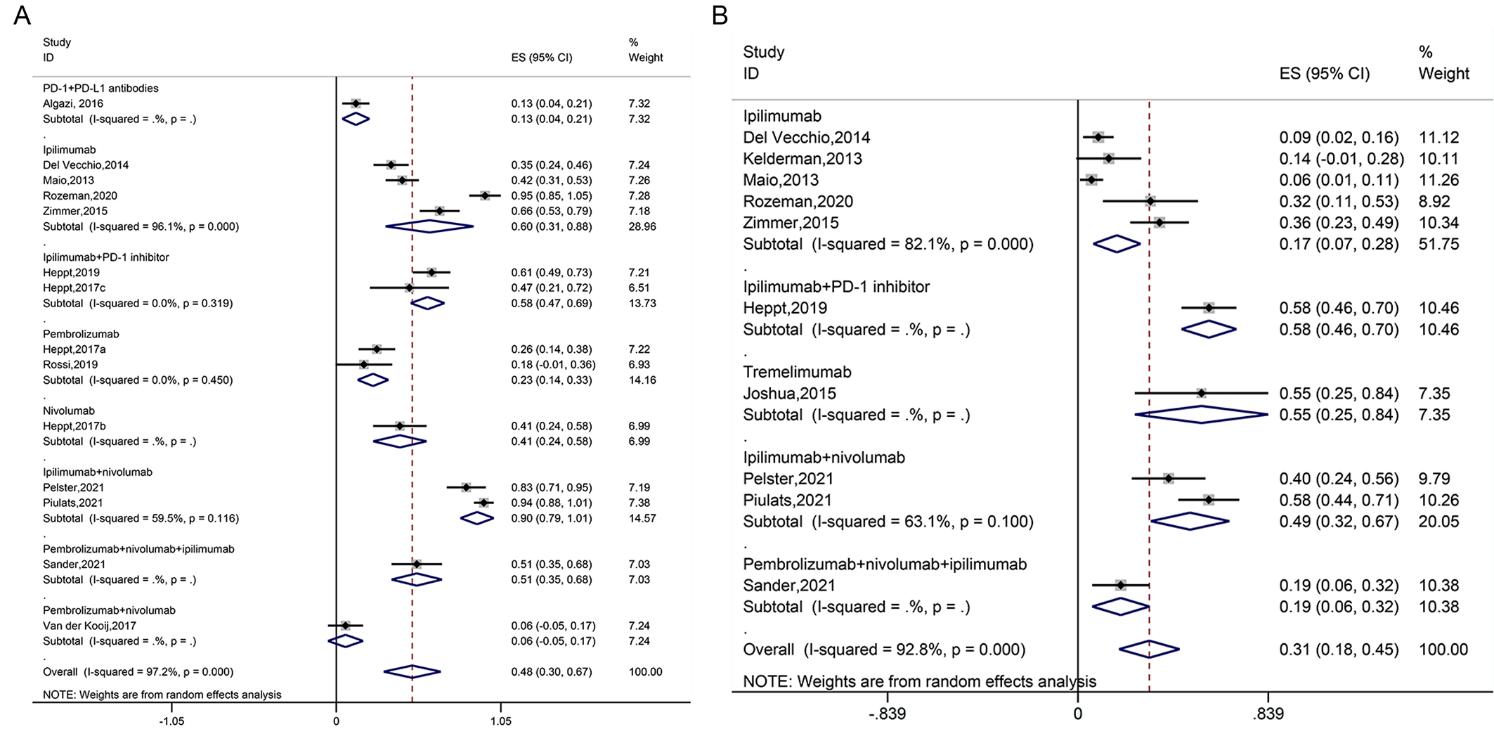
(A) Forest plot of adverse events (AEs) after treatment with immune checkpoint blockade about subgroups analysis. (B) Forest plot of serious adverse event (SAEs) after treatment with immune checkpoint blockade about subgroups analysis.

**Figure S4****.**


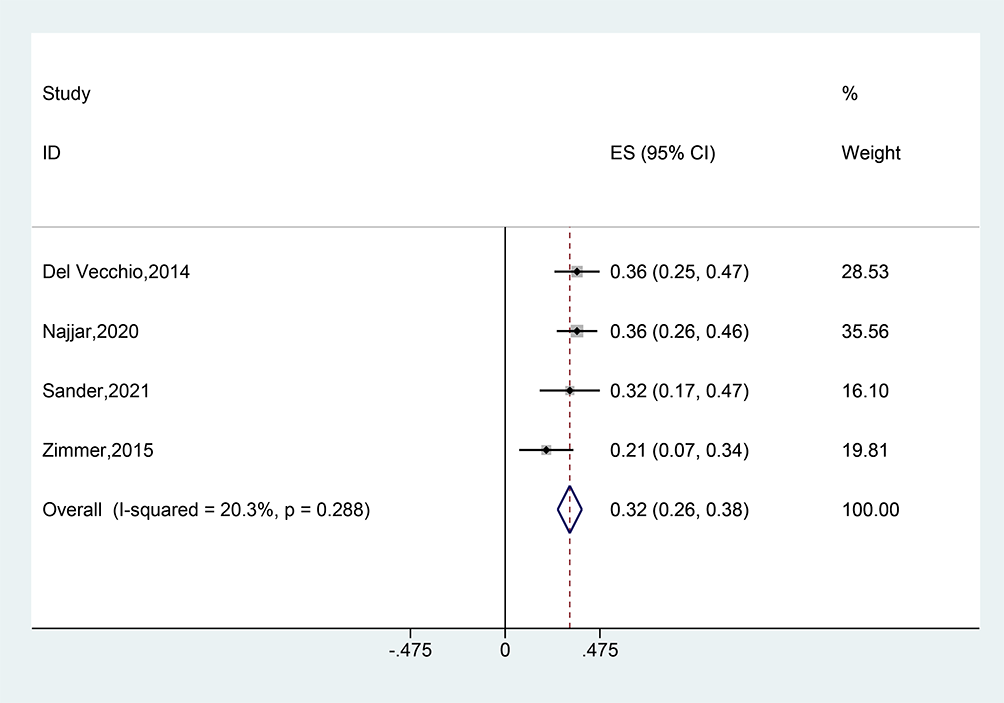


Forest plot of disease control rate (DCR) with immune checkpoint blockade treatment.

**Figure S5.**


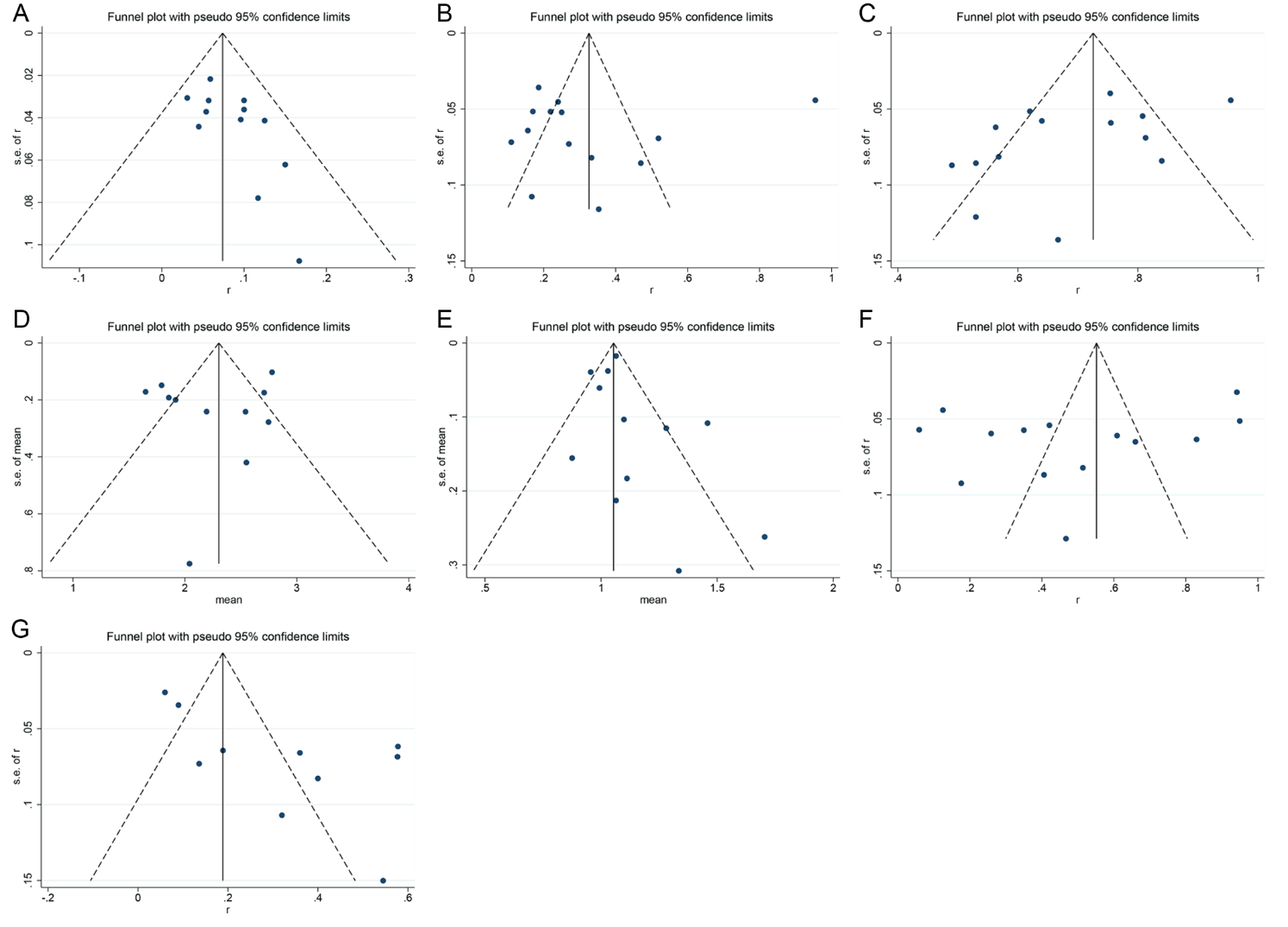


(A) Funnel plot for partial response (PR). (B) Funnel plot for stable disease (SD). (C) Funnel plot for progressive disease (PD). (D) Funnel plot for overall survival (OS). (E) Funnel plot for progression free survival (PFS) . (F) Funnel plot for adverse events (AEs). (G) Funnel plot for serious adverse event (SAEs).

**Figure S6.**


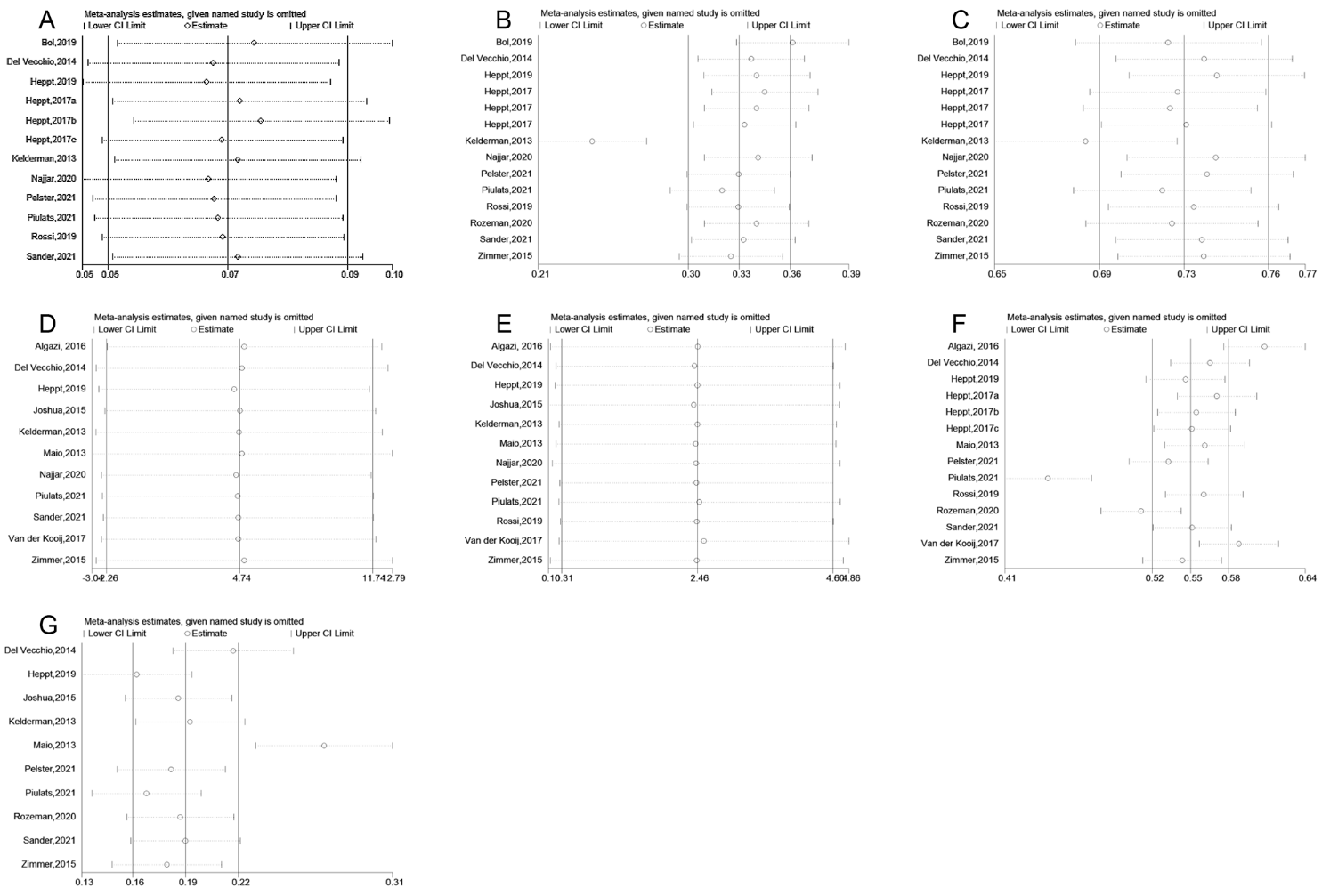


(A) Sensitivity analysis for partial response (PR). (B) Sensitivity analysis for stable disease (SD). (C) Sensitivity analysis for progressive disease (PD). (D) Sensitivity analysis for overall survival (OS). (E) Sensitivity analysis for progression free survival (PFS). (F) Sensitivity analysis for adverse events (AEs). (G) Sensitivity analysis for serious adverse event (SAEs).
